# Colonoscopy outcomes and clinical factors associated with significant findings in patients with positive fecal occult blood tests: A retrospective cohort study

**DOI:** 10.1097/MD.0000000000049414

**Published:** 2026-06-19

**Authors:** Mehmet Emin Gönüllü, Mehmet Fuat Çetin, Erman Yekenkurul, Fatih Gürsoy

**Affiliations:** a Department of General Surgery, Bilecik Şeyh Edebali University Faculty of Medicine, Bilecik, Türkiye; b Department of General Surgery, Duzce University Faculty of Medicine, Duzce, Türkiye.

**Keywords:** anemia, colonoscopy, colorectal neoplasms, fecal occult blood test, risk factors

## Abstract

Fecal occult blood test (FOBT) is widely used in colorectal cancer (CRC) screening; however, its ability to predict clinically significant pathology may vary across patient populations. This study aimed to evaluate colonoscopy outcomes in FOBT-positive patients and to identify clinical factors associated with clinically significant colonoscopic findings. This retrospective single-center cohort study included 216 adult patients with positive FOBT results who underwent colonoscopy between 2018 and 2024. Demographic characteristics, hemoglobin levels, FOBT methods, colonoscopic findings, and histopathological results were analyzed. Clinically significant pathology was defined as the presence of adenoma, advanced adenoma, CRC, or inflammatory lesions. Multivariable logistic regression analysis was performed to identify factors associated with clinically significant pathology, and receiver operating characteristic analysis was used to evaluate discriminative performance. Clinically significant pathology was detected in 57.4% of patients. The most frequent findings were adenomas (28.7%), advanced adenomas (14.8%), and CRC (8.3%). Older age (≥60 years), male sex, and anemia were significantly associated with clinically significant findings. Among these variables, anemia showed the strongest association with significant pathology. Receiver operating characteristic analysis demonstrated moderate discriminative ability for individual variables, whereas a combined model incorporating age, sex, and anemia achieved the highest performance (area under the curve = 0.81). FOBT positivity was associated with a high rate of clinically significant colonoscopic findings. Older age, male sex, and anemia may help identify higher-risk patients; however, these factors should not be used to exclude any FOBT-positive patient from colonoscopy. Prospective multicenter studies are needed to validate these findings.

## 1. Introduction

Colorectal cancer (CRC) is one of the most common malignancies worldwide and represents a major cause of cancer-related morbidity and mortality. According to the Global Cancer Statistics 2020, CRC ranks third in terms of incidence and second in cancer-related deaths globally.^[1,2]^ The prognosis of CRC is highly dependent on the stage at diagnosis: while early-stage disease is associated with favorable survival rates, advanced-stage CRC continues to carry a poor prognosis. These facts underscore the critical importance of effective screening strategies in enabling early detection and timely intervention.^[3,4]^

The fecal occult blood test (FOBT) has long been utilized as a cornerstone of CRC screening programs due to its noninvasive nature, low cost, and widespread availability. FOBT can be performed using guaiac-based methods or immunochemical techniques, the latter demonstrating superior sensitivity and specificity in detecting lower gastrointestinal bleeding.^[5,6]^ Numerous studies have reported that FOBT positivity may be associated with clinically significant lesions such as adenomatous polyps, advanced adenomas, and CRC. However, FOBT positivity does not invariably correlate with pathological findings, and a considerable proportion of patients undergo colonoscopy without clinically relevant outcomes.^[7–9]^

Colonoscopy remains the gold standard diagnostic and therapeutic tool in the evaluation of colorectal pathology. Performing colonoscopy in patients with a positive FOBT enables not only the detection of early-stage cancers but also the identification and removal of precancerous lesions.^[10–12]^ Nevertheless, the predictive value of FOBT positivity is influenced by several clinical factors, including age, sex, hemoglobin level, and the presence of anemia. Determining which subsets of FOBT-positive patients are most likely to harbor significant pathology remains an area of active clinical interest.^[13,14]^

In Türkiye, FOBT has been incorporated into the national CRC screening program as a first-line modality.^[15]^ Yet, questions remain regarding the proportion of FOBT-positive patients who present with clinically significant colonoscopic findings and the patient-related associated factors of such outcomes. Evaluating these relationships through local patient data is essential to improve the efficiency of screening strategies, minimize unnecessary colonoscopies, and optimize the prioritization of high-risk individuals.

Therefore, the present study aimed to evaluate the colonoscopy findings of patients with positive FOBT results and to determine the clinical factors associated with significant pathology in a single-center retrospective cohort. Secondary objectives included assessing the predictive role of variables such as age, sex, and anemia in the occurrence of advanced adenomas and CRC.

## 2. Materials and methods

### 2.1. Study design, setting, and ethical approval

This retrospective, single-center cohort study was conducted at the Duzce University Faculty of Medicine, Department of General Surgery. The medical records of patients with positive FOBT results between January 2018 and December 2024 were retrieved and analyzed using the institutional hospital information system. The study protocol was approved by the Duzce University Non-Interventional Clinical Research Ethics Committee (Approval number: 2025/214, Date: August 25, 2025) and was performed in accordance with the ethical standards of the Declaration of Helsinki. Owing to the retrospective design, the need for written informed consent was waived by the ethics committee.

### 2.2. Patient population

**Figure 1.**
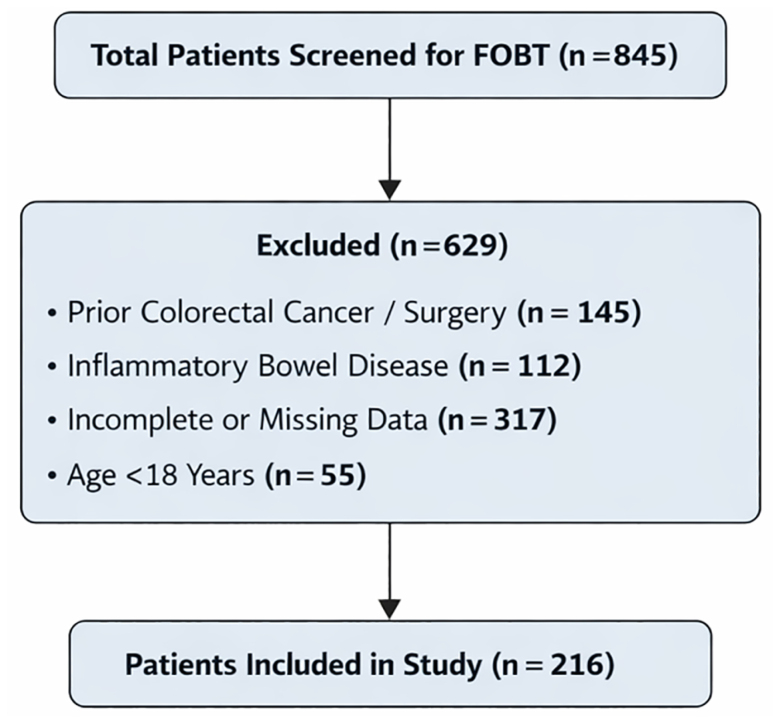
Flow diagram of patient selection process. FOBT = fecal occult blood test.

Eligible participants were adults aged 18 years or older who had a positive FOBT and underwent colonoscopy within 6 months of testing. Only patients with complete colonoscopy and pathology records were included. The exclusion criteria were: positive FOBT without subsequent colonoscopy, incomplete or missing colonoscopy data, previous history of CRC or major colorectal surgery, preexisting inflammatory bowel disease, and age under 18 years. A flow diagram illustrating the patient selection process, including the number of screened patients, excluded cases with reasons, and the final study population (*n* = 216), is presented in Figure 1. Potential selection bias cannot be excluded due to the retrospective design.

### 2.3. Fecal occult blood testing

FOBT was performed using both guaiac-based kits and immunochemical tests, depending on availability during the study period. Patients were instructed to collect 2 consecutive stool samples in sterile containers, which were then processed in the hospital’s central biochemistry laboratory according to manufacturer guidelines. Guaiac-based tests were interpreted as positive when at least one sample demonstrated a color change after the developer solution application. Immunochemical FOBTs were analyzed via automated immunoassay, and positivity was defined according to kit-specific cutoff values. The test method (guaiac vs immunochemical) and results were recorded for each patient.^[16]^ Dietary restrictions prior to guaiac-based testing could not be consistently verified due to the retrospective design.

### 2.4. Colonoscopy procedure

All colonoscopies were performed by board-certified gastroenterologists or colorectal surgeons using standard video colonoscopes (Olympus CF-HQ190L/I or equivalent models). All procedures were performed according to standard institutional protocols. Bowel preparation was achieved with a polyethylene glycol-based oral solution administered the day before the procedure. Conscious sedation with intravenous midazolam and/or propofol was applied in accordance with institutional protocols. The colon was intubated to the cecum in all cases unless contraindicated, and mucosal inspection was performed during both insertion and withdrawal phases.

Detected lesions were documented regarding their size, location, and macroscopic appearance. Polypectomy and biopsy specimens were obtained when indicated, and all samples were examined by experienced pathologists in the Department of Pathology using hematoxylin and eosin staining. Histological diagnoses were classified according to World Health Organization criteria for colorectal neoplasia.^[17]^

### 2.5. Data collection

Data were collected retrospectively from multiple modules of the hospital information system, including laboratory results (FOBT, hemoglobin), endoscopy reports (colonoscopy findings), and pathology records. Recorded variables included patient demographics (age, sex), hemoglobin level, presence of anemia (defined as hemoglobin <12 g/dL in women and <13 g/dL in men), FOBT method and result, colonoscopy findings, histopathological diagnoses, and the time interval between FOBT and colonoscopy. All patient identifiers were removed, and data were anonymized prior to analysis.

### 2.6. Classification of colonoscopy findings

Colonoscopy findings were categorized as normal mucosa, adenomatous polyp, advanced adenoma (defined as size ≥10 mm, villous or tubulovillous histology, or presence of high-grade dysplasia), CRC, inflammatory lesions (such as colitis or ulcerative lesions), and other findings including diverticulosis, hemorrhoids, or minor mucosal changes. For analytical purposes, all patients were further stratified into 2 groups: those with clinically significant pathology, defined as adenoma, advanced adenoma, CRC, or inflammatory lesions, and those without significant pathology, including normal colonoscopy, diverticulosis, hemorrhoids, or minor mucosal abnormalities.

### 2.7. Statistical analysis

All statistical analyses were performed using SPSS version 25.0 (IBM Corp., Armonk, NY, USA). Continuous variables were expressed as mean ± standard deviation or median (minimum–maximum) according to their distribution, while categorical variables were presented as frequency and percentage.

The Kolmogorov–Smirnov test was used to evaluate the normality of the distribution. For comparisons between groups with and without clinically significant pathology, the independent samples *t* test was applied for normally distributed continuous variables, while the Mann–Whitney *U* test was used for non-normally distributed variables. Chi-square test or Fisher’s exact test was used to compare categorical variables.

To identify independently associated factors of clinically significant pathology, multivariate logistic regression analysis was performed. Variables with *P* < .10 in univariate analysis were included in the regression model, and results were reported as odds ratios (ORs) with 95% confidence intervals (CIs).

The predictive accuracy of significant variables (age, sex, anemia) for advanced adenoma or CRC was assessed using receiver operating characteristic (ROC) curve analysis. Area under the curve (AUC) values, sensitivity, specificity, and optimal cutoff points were reported.

All statistical tests were two-tailed, and a *P*-value < .05 was considered statistically significant.

A post hoc power analysis was performed using G*Power 3.1 software. Based on the observed effect sizes in this study, the required minimum sample size was calculated as 168 patients to achieve 80% statistical power at a 5% significance level. Since the study included 216 patients, the sample size was deemed sufficient to ensure adequate statistical power.^[18]^

## 3. Results

According to Table 1, a total of 216 patients with positive FOBTs were included in the study. The mean age of patients with clinically significant pathology was 61.8 ± 9.7 years, which was significantly higher than those without significant pathology (55.3 ± 10.1 years, *P* = .002). Male sex was more frequent in the pathology-positive group (62.9% vs 44.6%, *P* = .014). The mean hemoglobin level was lower in patients with significant pathology (11.9 ± 1.8 g/dL) compared to the pathology-negative group (13.4 ± 1.6 g/dL, *P* < .001). Similarly, anemia was more common among patients with significant pathology (63.7% vs 30.4%, *P* < .001).

**Table 1:** Demographic and clinical characteristics of the patients (n = 216).

Variable	Clinically significant pathology (+) n = 124	Clinically significant pathology (–) n = 92	*P*-value
Age (yr, mean ± SD)	61.8 ± 9.7	55.3 ± 10.1	.002
Male sex (%)	78 (62.9)	41 (44.6)	.014
Hemoglobin (g/dL, mean ± SD)	11.9 ± 1.8	13.4 ± 1.6	<.001
Anemia (%)	79 (63.7)	28 (30.4)	<.001
FOBT method (Immunochemical/Guaiac)	92/ 32	74/ 18	.273

FOBT *=* fecal occult blood test, SD *=* standard deviation.

According to Table 2, adenomatous polyps were the most common finding (28.7%), followed by advanced adenomas (14.8%) and CRC (8.3%). A total of 25.9% of patients had normal colonoscopy results, while inflammatory lesions and other findings such as diverticulosis or hemorrhoids were observed in 5.6% and 16.7% of cases, respectively.

**Table 2:** Distribution of colonoscopy findings (n = 216).

Colonoscopy findings	*n*	%
Normal	56	25.9
Adenomatous polyp	62	28.7
Advanced adenoma	32	14.8
Colorectal cancer	18	8.3
Inflammatory lesions	12	5.6
Other (diverticulosis, hemorrhoids, etc)	36	16.7

According to Table 3, clinically significant pathology was more common in patients aged ≥60 years (67.7% vs 41.3%, *P* < .001), in males (62.9% vs 44.6%, *P* = .014), and in those with anemia (63.7% vs 30.4%, *P* < .001). No significant association was found between FOBT method and the presence of significant pathology (74.2% vs 80.4%, *P* = .273).

**Table 3:** Factors associated with clinically significant pathology.

Variable	Significant pathology (+) n = 124	Significant pathology (–) n = 92	*P*-value
Age ≥60 yr (%)	84 (67.7)	38 (41.3)	<.001
Male sex (%)	78 (62.9)	41 (44.6)	.014
Hemoglobin < 12 g/dL (%)	79 (63.7)	28 (30.4)	<.001
Immunochemical FOBT (%)	92 (74.2)	74 (80.4)	.273

FOBT = fecal occult blood test.

According to Table 4, multivariate logistic regression analysis showed that age ≥60 years was associated with a 2.48-fold increased risk of clinically significant pathology (95% CI: 1.40–4.40, *P* = .002). Male sex was associated with a nearly two-fold increased risk (OR = 1.91, 95% CI: 1.11–3.30, *P* = .020), while anemia was the strongest independently associated factor, with an almost four-fold increased risk (OR = 3.87, 95% CI: 2.08–7.18, *P* < .001).

**Table 4:** Logistic regression analysis for clinically significant pathology.

Variable	OR	95% CI	*P*-value
Age ≥60 yr	2.48	1.40–4.40	.002
Male sex	1.91	1.11–3.30	.020
Anemia	3.87	2.08–7.18	<.001

CI *=* confidence interval, OR *=* odds ratio.

According to Table 5, ROC curve analysis showed that anemia had the highest predictive accuracy for advanced adenoma and CRC (AUC = 0.74, 95% CI: 0.67–0.81, *P* < .001; sensitivity 72%, specificity 70%). Age ≥60 years had moderate discriminatory value (AUC = 0.67, 95% CI: 0.59–0.75, *P* = .001; sensitivity 68%, specificity 61%), while male sex had limited predictive power (AUC = 0.58, 95% CI: 0.50–0.66, *P* = .045; sensitivity 63%, specificity 55%). The combined model including age, sex, and anemia demonstrated the highest discriminatory performance (AUC = 0.81, 95% CI: 0.75–0.87, *P* < .001; sensitivity 78%, specificity 73%).

**Table 5:** ROC analysis for prediction of advanced adenoma or colorectal cancer.

Parameter	AUC	95% CI	*P*-value	Sensitivity (%)	Specificity (%)
Age ≥60 yr	0.67	0.59–0.75	.001	68	61
Male sex	0.58	0.50–0.66	.045	63	55
Anemia	0.74	0.67–0.81	<.001	72	70
Combined model (age + sex + anemia)	0.81	0.75–0.87	<.001	78	73

AUC *=* area under the curve, CI *=* confidence interval.

## 4. Discussion

In this retrospective single-center study, we evaluated colonoscopy outcomes in patients with positive FOBT and investigated clinical associated factors of clinically significant pathology. Among 216 patients, more than half (57.4%) were found to have significant lesions, including adenomas, advanced adenomas, CRC, and inflammatory findings. Both logistic regression and ROC analyses demonstrated that older age, male sex, and the presence of anemia were independently associated factors of significant pathology.

In our cohort, patients aged ≥60 years were at increased risk of clinically significant pathology (OR = 2.48, 95% CI: 1.40–4.40, *P* = .002). ROC analysis further confirmed the moderate discriminatory value of age (AUC = 0.67, 95% CI: 0.59–0.75, *P* = .001; sensitivity 68%, specificity 61%). These findings align with prior reports suggesting that the predictive value of FOBT increases with age.^[11,19,20]^ Mousavinezhad et al demonstrated that neoplasia detection rates were higher in older subgroups of FOBT-positive patients, although age alone was not sufficient as a sole predictive marker.^[21]^

Male sex was also associated with nearly a two-fold increased risk of significant pathology (OR = 1.91, 95% CI: 1.11–3.30, *P* = .020). However, its stand-alone discriminatory capacity was limited (AUC = 0.58, *P* = .045). This observation is consistent with previous studies, which have shown higher rates of adenomatous polyps and CRC in men compared with women.^[21,22]^ While biological differences, lifestyle factors, and screening participation rates may contribute, the exact mechanisms underlying these sex-based disparities remain uncertain.

The strongest association observed in our study was related to anemia (OR = 3.87, 95% CI: 2.08–7.18, *P* < .001). ROC analysis also demonstrated that anemia had the highest discriminative performance among the evaluated variables. These findings are consistent with previous reports indicating that anemia, particularly iron-deficiency anemia, is closely linked to colorectal malignancy.^[23]^ Muinuddin et al reported that the coexistence of FOBT positivity and anemia substantially increased the likelihood of malignant findings on colonoscopy.^[24]^ Our results suggest that hemoglobin levels may serve as a useful clinical marker in the risk assessment of FOBT-positive patients.^[25,26]^ Importantly, this relationship should be interpreted with caution, as anemia may reflect underlying gastrointestinal bleeding rather than representing a causal predictive factor, introducing the possibility of protopathic bias.

In our analysis, there was no significant difference in colonoscopic yield between guaiac-based and immunochemical tests (*P* = .273). Nonetheless, immunochemical FOBT has been widely reported to demonstrate superior sensitivity and specificity compared with guaiac-based methods, particularly in large-scale screening programs. van der Vlugt et al.^[27]^ and others have emphasized higher participation rates and improved neoplasia detection with immunochemical testing.^[28]^ The lack of significant difference in our study may be attributable to the limited sample size and the retrospective nature of data collection.

Importantly, the combined model incorporating age, sex, and anemia demonstrated the highest predictive power (AUC = 0.81, 95% CI: 0.75–0.87, *P* < .001; sensitivity 78%, specificity 73%), outperforming individual parameters. This supports the concept that multifactorial risk stratification may improve the efficiency of CRC screening programs. Previous reports have also suggested that composite models provide superior predictive value compared with single risk factors alone.

Our findings suggest that FOBT-positive patients who are older, male, and anemic represent a subgroup at higher-risk of clinically significant pathology. While colonoscopy remains the gold standard for all FOBT-positive patients, these variables may inform prioritization strategies, particularly in healthcare systems with limited endoscopic capacity. Nevertheless, caution is warranted, as a considerable proportion of FOBT-positive patients without these risk factors were also found to harbor advanced adenomas or cancer.

Importantly, the observed association between anemia and clinically significant pathology should be interpreted with caution. Anemia may reflect underlying gastrointestinal bleeding rather than representing a causal factor, particularly in the context of colorectal neoplasia. Therefore, anemia should be considered a clinically relevant marker associated with disease presence rather than a definitive predictive factor.

### 4.1. Study limitations

Several limitations of this study must be acknowledged. First, the retrospective design limited data availability to existing hospital records, and some potentially relevant confounding variables, such as family history, lifestyle habits, and dietary factors, could not be evaluated. Second, as a single-center study, the generalizability of the findings may be limited to similar healthcare settings. Third, the use of both guaiac-based and immunochemical FOBTs may have introduced variability, given their known differences in sensitivity and specificity. In addition, dietary restrictions prior to guaiac-based testing could not be consistently verified due to the retrospective nature of the study, which may have influenced test positivity rates. Fourth, anemia, although strongly associated with clinically significant findings, may represent an underlying manifestation of gastrointestinal pathology rather than an independent predictive factor, introducing the possibility of protopathic bias. Finally, colonoscopy-related factors, including bowel preparation quality and inter-operator variability, were not assessed and may have influenced lesion detection rates.

## 5. Conclusion

In conclusion, this study demonstrates that FOBT positivity is associated with a substantial rate of clinically significant pathology. Older age, male sex, and anemia were found to be strongly associated with significant findings, with anemia showing the highest discriminative performance. A combined model incorporating these variables provided improved accuracy in identifying patients at higher risk of advanced lesions. These findings may support risk stratification in FOBT-positive patients, particularly in settings with limited endoscopic resources. However, these factors should be interpreted as clinical markers rather than definitive predictors, as anemia may reflect underlying gastrointestinal pathology rather than a causal determinant. Importantly, even patients with a lower-risk profile may still harbor clinically significant lesions, and therefore, colonoscopy remains essential for all FOBT-positive individuals. Further multicenter, prospective studies with larger cohorts are needed to validate these findings and refine clinical decision-making strategies.

## Author contributions

**Conceptualization:** Mehmet Emin Gönüllü, Mehmet Fuat Çetin, Erman Yekenkurul, Fatih Gürsoy

**Data curation:** Mehmet Emin Gönüllü, Mehmet Fuat Çetin, Erman Yekenkurul, Fatih Gürsoy.

**Formal analysis:** Mehmet Emin Gönüllü, Mehmet Fuat Çetin, Erman Yekenkurul, Fatih Gürsoy.

**Funding acquisition:** Mehmet Emin Gönüllü, Mehmet Fuat Çetin, Erman Yekenkurul, Fatih Gürsoy.

**Investigation:** Mehmet Emin Gönüllü, Mehmet Fuat Çetin, Erman Yekenkurul, Fatih Gürsoy.

**Methodology:** Mehmet Emin Gönüllü, Mehmet Fuat Çetin, Erman Yekenkurul, Fatih Gürsoy.

**Project administration:** Mehmet Emin Gönüllü, Mehmet Fuat Çetin, Erman Yekenkurul, Fatih Gürsoy.

**Resources:** Mehmet Emin Gönüllü, Mehmet Fuat Çetin, Fatih Gürsoy.

**Software:** Mehmet Emin Gönüllü, Mehmet Fuat Çetin, Fatih Gürsoy.

**Supervision:** Mehmet Emin Gönüllü, Mehmet Fuat Çetin, Fatih Gürsoy.

**Validation:** Mehmet Emin Gönüllü, Mehmet Fuat Çetin, Erman Yekenkurul, Fatih Gürsoy.

**Visualization:** Mehmet Emin Gönüllü, Mehmet Fuat Çetin, Erman Yekenkurul, Fatih Gürsoy.

**Writing – original draft:** Mehmet Emin Gönüllü, Mehmet Fuat Çetin, Erman Yekenkurul, Fatih Gürsoy.

## References

[1] Morgan E Arnold M Gini A. Global burden of colorectal cancer in 2020 and 2040: incidence and mortality estimates from GLOBOCAN. Gut. 2023;72:338–44. 36604116 10.1136/gutjnl-2022-327736

[2] Sung H Ferlay J Siegel RL. Global cancer statistics 2020: GLOBOCAN estimates of incidence and mortality worldwide for 36 cancers in 185 countries. CA Cancer J Clin. 2021;71:209–49. 33538338 10.3322/caac.21660

[3] Li J Li ZP Ruan WJ Wang W. Colorectal cancer screening: the value of early detection and modern challenges. World J Gastroenterol. 2024;30:2726–30. 38855153 10.3748/wjg.v30.i20.2726 PMC11154673

[4] Rawla P Sunkara T Barsouk A. Epidemiology of colorectal cancer: incidence, mortality, survival, and risk factors. Przeglad Gastroenterol. 2019;14:89–103. 10.5114/pg.2018.81072 PMC6791134 31616522

[5] Şahin TA Güzel EC. How effective is a fecal occult blood test to detect malignancy? Namik Kemal Med J. 2022;10:95–100.

[6] Li JN Yuan SY. Fecal occult blood test in colorectal cancer screening. J Dig Dis. 2019;20:62–4. 30714325 10.1111/1751-2980.12712

[7] Esmer AC Yeğen C. Fecal occult blood test, is it still worth for colorectal cancer screening? Polski Przeglad Chirurgiczny. 2022;95:1–5. 10.5604/01.3001.0015.9661 36805995

[8] Yuan SY Wu W Fu J. Quantitative immunochemical fecal occult blood test for neoplasia in colon cancer screening. J Dig Dis. 2019;20:78–82. 30714346 10.1111/1751-2980.12711

[9] Cobo-Cuenca AI Laredo-Aguilera JA Rodríguez-Borrego MA Santacruz-Salas E Carmona-Torres JM. Temporal trends in fecal occult blood test: associated factors (2009-2017). Int J Environ Res Public Health. 2019;16:5014. 31207996 10.3390/ijerph16122120 PMC6616453

[10] Issa IA Noureddine M. Colorectal cancer screening: an updated review of the available options. World J Gastroenterol. 2017;23:5086–96. 28811705 10.3748/wjg.v23.i28.5086 PMC5537177

[11] Mayir B Ensari C Durhan A Çöpelçi Y. Colonoscopy findings in patients who have positive fecal occult blood test for colorectal cancer screening. Turk J Colorectal Dis. 2018;28:27–30.

[12] Yücel M Demirpolat MT Yildirak MK. Colorectal cancer screening; colonoscopy and biopsy results in people undergoing colonoscopy due to positive fecal occult blood test. Tur J Surg. 2024;40:59–64. 10.47717/turkjsurg.2024.6352 PMC11257727 39036003

[13] Chang JY Moon CM Shim KN. Positive fecal occult blood test is a predictive factor for gastrointestinal bleeding after capsule endoscopy in patients with unexplained iron deficiency anemia: a Korean multicenter CAPENTRY Study. Clin Endoscopy. 2020;53:719–26. 10.5946/ce.2019.149 PMC7719424 33153246

[14] Challa SR Buluku G Gboluaje T. Assessment of fecal occult blood testing in acute hospital settings: perspectives of internal medicine residents in a multicenter study. Cureus. 2025;17:e88524. 40861722 10.7759/cureus.88524 PMC12372565

[15] Aydogan Gedik S Metintas S Onsuz MF. Recognition and participation of colorectal cancer screening in Turkiye: a systematic review and meta-analysis study. Northern Clin Istanbul. 2023;10:819–29. 10.14744/nci.2022.94103 PMC10846582 38328722

[16] Li S Wang H Hu J. New immunochemical fecal occult blood test with two-consecutive stool sample testing is a cost-effective approach for colon cancer screening: results of a prospective multicenter study in Chinese patients. Int J Cancer. 2006;118:3078–83. 16425283 10.1002/ijc.21774

[17] Latos W Aebisher D Latos M. Colonoscopy: preparation and potential complications. Diagnostics (Basel, Switzerland). 2022;12:747. 35328300 10.3390/diagnostics12030747 PMC8947288

[18] Liang PS Zaman A Kaminsky A. Blood test increases colorectal cancer screening in persons who declined colonoscopy and fecal immunochemical test: a randomized controlled trial. Clin Gastroenterol Hepatol. 2023;21:2951–7.e2. 37037262 10.1016/j.cgh.2023.03.036 PMC10523873

[19] Miglioretti DL Rutter CM Bradford SC. Improvement in the diagnostic evaluation of a positive fecal occult blood test in an integrated health care organization. Med Care. 2008;46(9 Suppl 1):S91–96. 18725839 10.1097/MLR.0b013e31817946c8 PMC4227983

[20] Pioche M Ganne C Gincul R. Colon capsule versus computed tomography colonography for colorectal cancer screening in patients with positive fecal occult blood test who refuse colonoscopy: a randomized trial. Endoscopy. 2018;50:761–9. 29486502 10.1055/s-0044-100721

[21] Mousavinezhad M Majdzadeh R Akbari Sari A Delavari A Mohtasham F. The effectiveness of FOBT vs. FIT: a meta-analysis on colorectal cancer screening test. Med J Islamic Republic Iran. 2016;30:366. PMC4972062 27493910

[22] Tsokkou S Konstantinidis I Papakonstantinou M. Sex differences in colorectal cancer: epidemiology, risk factors, and clinical outcomes. J Clin Med. 2025;14:5539. 40807160 10.3390/jcm14155539 PMC12347225

[23] Chardalias L Papaconstantinou I Gklavas A Politou M Theodosopoulos T. Iron deficiency anemia in colorectal cancer patients: is preoperative intravenous iron infusion indicated? A narrative review of the literature. Cancer Diagnosis Prognosis. 2023;3:163–8. 36875314 10.21873/cdp.10196 PMC9949551

[24] Muinuddin A Aslahi R Hopman WM Paterson WG. Relationship between the number of positive fecal occult blood tests and the diagnostic yield of colonoscopy. Can J Gastroenterol. 2013;27:90–4. 23472244 10.1155/2013/612314 PMC3731119

[25] Nakama H Zhang B Fattah AS Zhang X. Colorectal cancer in iron deficiency anemia with a positive result on immunochemical fecal occult blood. Int J Colorectal Dis. 2000;15:271–4. 11151429 10.1007/s003840000255

[26] Méndez G Rivera-Matos L Shuja A. Faecal occult blood testing: a review of its use and common misutilisation. BMJ Open Gastroenterol. 2025;12:e001876. 10.1136/bmjgast-2025-001876 PMC12273078 40675631

[27] van der Vlugt M Grobbee EJ Bossuyt PMM. Interval colorectal cancer incidence among subjects undergoing multiple rounds of fecal immunochemical testing. Gastroenterology. 2017;153:439–47.e2. 28483499 10.1053/j.gastro.2017.05.004

[28] Guittet L Bouvier V Mariotte N. Comparison of a guaiac based and an immunochemical faecal occult blood test in screening for colorectal cancer in a general average risk population. Gut. 2007;56:210–4. 16891354 10.1136/gut.2006.101428 PMC1856766

